# Review of "Ethics in Mental Health Research" by James M. DuBois

**DOI:** 10.1186/1747-5341-3-11

**Published:** 2008-04-03

**Authors:** Ronald Pies

**Affiliations:** 1 Department of Psychiatry, S.U.N.Y. Upstate Medical University, Syracuse, N.Y. 13210, USA; 2 Department of Psychiatry, Tufts University School of Medicine, Boston, MA, 02111, USA

## Abstract

The burgeoning field of medical ethics raises complicated questions for mental health researchers. The critical issues of risk assessment, beneficence, and the moral duties researchers owe their patients are analyzed in James DuBois's well written *Ethics in Mental Health Research*.

## Review

*Only one rule in medical ethics need concern you – that action on your part which best conserves the interests of your patient. ~Martin H. Fischer *[1]

*I wondher why ye can always read a doctor's bill an' ye niver can read his purscription. ~Finley Peter Dunne *[2]

I recall, many years ago, how I wanted to mail a research questionnaire to the general community, on an issue in mental health. As a faculty member of a large Boston teaching hospital, I was surprised to learn that my "simple little questionnaire" had to undergo extensive evaluation by our institutional review board (IRB). *How*, I was asked, *might this questionnaire affect a recipient's mental health? Would the shock of receiving it in the mail cause some people to become anxious or depressed? How would I protect the confidentiality of those who responded, even though the forms did not require any names? *(Perhaps, the IRB opined, some subjects could be identified on the basis of their demographics). I considered our IRB's concerns fussy and obsessive, and to this day, I still do. And yet, examined from within the moral framework constructed by Prof. James M. Dubois in his excellent new *Ethics in Mental Health Research*, I can understand (sort of) why our IRB raised these issues.

Prof. DuBois, the Chair of the Department of Health Ethics at Saint Louis University, is aware that special issues arise when discussing research on those diagnosed with mental illness. Although the book appears aimed at "mental health researchers, IRB members, and research advocates," I believe it will be of interest to most physicians and mental health professionals who struggle with issues in medical ethics. As Prof. DuBois shows with admirable clarity, there are rarely simple or easy answers to the conundrums that arise in these fields. He therefore advocates a "balanced approach" to research ethics, realizing that while the rights of mentally ill persons must be protected, "...research holds an important key to improving the lives of people who suffer from mental disorders." (p. 5).

In ten well-written chapters – remarkably, all penned by Prof. DuBois – the entire range of topics in mental health research is covered. The first three chapters develop theoretical foundations for research ethics, including a splendid chapter entitled "An Ethical Framework for Research." Focusing on the Belmont Report (1976–78), DuBois lays out the three cardinal principles of research ethics: *respect for persons; beneficence*, and *justice*. The last principle has to do with "...the distribution not only of the benefits of research...but also the burdens of participation in research." (pp. 28–29). Chapter three provides a useful framework for addressing ethical dilemmas and "..balancing competing goods and principles". The remainder of the book deals with "applied" topics, such as informed consent; decision-making capacity; risk-benefit analysis; participant recruitment; privacy and confidentiality; and conflicts of interest. This last chapter is particularly helpful and practical, even as it reveals an array of ethical minefields awaiting the unwary clinician.

Prof. Dubois carefully avoids either pontificating or providing legal advice. Rather, he approaches the dilemmas of research ethics analytically, with numerous engaging case vignettes followed by DuBois' own commentary. These vignettes and their discussions do not "solve" the dilemmas posed; rather, they provide an analytical framework within which the researcher may understand and confront these conundrums.

DuBois is acutely aware of how language enters into debates about medical ethics, and spends time discussing the various constituencies and vested interests behind terms like "patient", "consumer", "client", and "mental disorder". Physicians will be pleased that, in general, they are not relegated to the Orwellian category of "providers" (a term that always conjures up someone in a white coat placing a food pellet into the mouth of a lab animal). Also evident throughout the book is a sense of fair-mindedness and humane values: DuBois is not one to demonize, even though the mental health field comes in for some harsh words in the introduction to the book. (The allusion to "the lack of successful treatments" (p. 4) in mental health care is both gratuitously insulting and factually inaccurate, notwithstanding the over-selling of some modern-day pharmaco-therapies. Lithium, electroconvulsive therapy, and cognitive-behavioral therapy are all examples of remarkably successful treatments, despite the misapplication of ECT in the early days of its use).

Such quibbles aside, I believe that Prof. DuBois' *Ethics in Mental Health Research *will set the standard for reasoned discussion of mental health research, and the moral dilemmas that arise therein. Although I would have liked more material specifically relating to psychiatrists and other physicians, I found much food for thought in the case vignettes. I believe that any mental health professional contemplating a research project would be remiss if he or she did not consult this wise and well-reasoned book.

## About the author

Ronald Pies MD is Professor of Psychiatry and Lecturer on Bioethics and Humanities at S.U.N.Y. Upstate Medical University in Syracuse, New York, and Clinical Professor of Psychiatry at Tufts University School of Medicine in Boston, Massachusetts. He is the author of *The Ethics of the Sages *(Rowman & Littlefield) and *Everything Has Two Handles: The Stoic's Guide to the Art of Living *(Hamilton Books) as well as several textbooks on psychopharmacology. He is interested in the connection between mental health care and various spiritual traditions.

## Competing interests

The author(s) declare that they have no competing interests.
